# Significant effect of HIV/HAART on oral microbiota using multivariate analysis

**DOI:** 10.1038/s41598-019-55703-9

**Published:** 2019-12-27

**Authors:** Ann L. Griffen, Zachary A. Thompson, Clifford J. Beall, Elizabeth A. Lilly, Carolina Granada, Kelly D. Treas, Kenneth R. DuBois, Shahr B. Hashmi, Chiranjit Mukherjee, Aubrey E. Gilliland, Jose A. Vazquez, Michael E. Hagensee, Eugene J. Leys, Paul L. Fidel

**Affiliations:** 1 Division of Pediatric Dentistry, The Ohio State University College of Dentistry, Columbus, OH USA; 2 Division of Biosciences, The Ohio State University College of Dentistry, Columbus, OH USA; 3 Center of Excellence in Oral and Craniofacial Biology, Louisiana State University Health Sciences Center School of Dentistry, New Orleans, LA USA; 4 Division of Infectious Diseases, Department of Medicine, Medical College of Georgia/Augusta University, Augusta, GA USA; 5 Section of Infectious Disease, Department of Medicine, Louisiana State University Health Sciences Center, New Orleans, LA USA

**Keywords:** Metagenomics, Microbiome

## Abstract

Persons infected with HIV are particularly vulnerable to a variety of oral microbial diseases. Although various study designs and detection approaches have been used to compare the oral microbiota of HIV-negative and HIV-positive persons, both with and without highly active antiretroviral therapy (HAART), methods have varied, and results have not been consistent or conclusive. The purpose of the present study was to compare the oral bacterial community composition in HIV-positive persons under HAART to an HIV-negative group using 16S rRNA gene sequence analysis. Extensive clinical data was collected, and efforts were made to balance the groups on clinical variables to minimize confounding. Multivariate analysis was used to assess the independent contribution of HIV status. Eighty-nine HIV-negative participants and 252 HIV-positive participants under HAART were sampled. The independent effect of HIV under HAART on the oral microbiome was statistically significant, but smaller than the effect of gingivitis, periodontal disease, smoking, caries, and other clinical variables. In conclusion, a multivariate comparison of a large sample of persons with HIV under HAART to an HIV-negative control group showed a complex set of clinical features that influenced oral bacterial community composition, including the presence of HIV under HAART.

## Introduction

HIV disease continues to be a life-threatening and economically important disease in the US and worldwide. Persons infected with HIV are particularly vulnerable to a wide variety of oral diseases including gingivitis, periodontitis, dental caries, endodontic infections, oropharyngeal candidiasis (OPC), necrotizing ulcerative periodontitis, oral warts, oral hairy leukoplakia, and Kaposi’s sarcoma^1–3^. The role of the oral microbiota has long been recognized in the etiology of many of these diseases. HIV infection and the related loss of CD4 cells can disrupt the balance between host immunity and the endogenous microbiota, potentially leading to significant shifts in the composition and diversity of bacterial communities. These shifts in microbiota may further impact the health of the oral tissue and local immunity, leading to increased susceptibility to pathogenic microbes and contributing to HIV-associated oral diseases. However, despite a number of studies, it is not entirely clear to what extent HIV infection and its related immune suppression and treatments impact the oral microbiota. Various study designs and detection methods have been used to compare the microbiota of saliva or subgingival samples in HIV-negative and HIV-positive subjects, both with and without highly active antiretroviral therapy (HAART)^2,4–17^. Some have shown small differences^5,6,9,11–13,18^, and some, no differences^2,4,16,17^. The largest differences have been observed between HIV-negative groups and untreated HIV-positive groups, with HAART tending to restore a microbiota closer to that of HIV-negative groups^5,15^. Limitations of these studies variously include small sample sizes, targeted assays that survey only a small subset of the total bacterial community, approaches not capable of resolving at the species level, and lack of accounting for potential clinical confounders such as demographic or health factors. Only 3 studies used current methods that allow comprehensive bacterial community composition analysis at the level of species^10,14,16^. Of these, only one involved a large sample size, but the participants were children with perinatally acquired HIV^16^, and it is unclear if results would generalize to adult-acquired disease. Consistent findings have not emerged from this body of work, and taken together these studies are inconclusive.

Viral load^19^, CD4 counts^8,9,14^, periodontal status^7,8,20^, and smoking^21^ have all been shown to be significant predictors of microbial composition in HIV-positive cohorts. If groups are not balanced, underlying differences among groups on these variables could easily obscure differences that result from HIV status, or lead to misattribution of differences that were not the result of HIV status. This suggests that a more comprehensive analytic approach that can separate the effects of other clinical variables is needed to sort out the contribution of HIV to the oral microbiome. Care is especially required for clinical variables known to account for relatively large differences, such as periodontal status, caries and smoking. Although they are all recognized as potential confounders, it is nearly impossible to simultaneously balance them perfectly in observational studies. Multivariate approaches have long been used to sort out the independent contributions of confounders, but have not been widely used in microbiome studies. Approaches that can be applied to high-dimensional microbiome data^22^ now make it possible to consider the effects of multiple clinical variables at the same time.

The purpose of the present study was to compare the oral microbial community composition in HIV-positive persons under HAART to an HIV-negative group using 16S rRNA gene sequence analysis. Care was taken to minimize potential confounders, and multivariate analysis was used to assess the independent contribution of HIV status. Using this approach, a significant independent effect of HIV treated with HAART on the oral microbiome was shown, but it was smaller than the effect of other clinical variables, including periodontal disease, smoking and caries.

## Results

**Figure 1:**
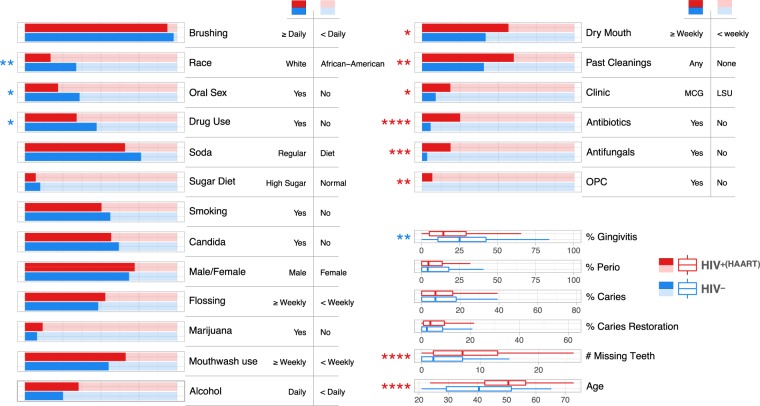
Balance for secondary clinical variables between the HIV positive and negative groups. Stacked bar graphs compare categorical variables for HIV^+(HAART)^ and HIV^−^ groups. Dichotomous categories are shown to the right of the labels. Significance was determined by Fisher’s exact test. Box and whisker plots of distributions for the HIV groups are shown for continuous variables. Significance was determined by Wilcoxon rank sum test. Asterisks indicate levels of significance: *p < 0.05; **p < 0.01; ***p < 0.001; ****p < 0.0001.

Eighty-nine HIV-negative subjects and 252 HIV-positive subjects under HAART were sampled. All HIV-positive subjects had been on antiretroviral therapy for a minimum of 180 days. The two groups were recruited to achieve balance on sex, caries and periodontitis. Secondary variables for the two cohorts are summarized in Fig. 1. A number of secondary clinical variables were significantly different by HIV status as shown, and interactions were considered in subsequent analyses.

**Figure 2:**
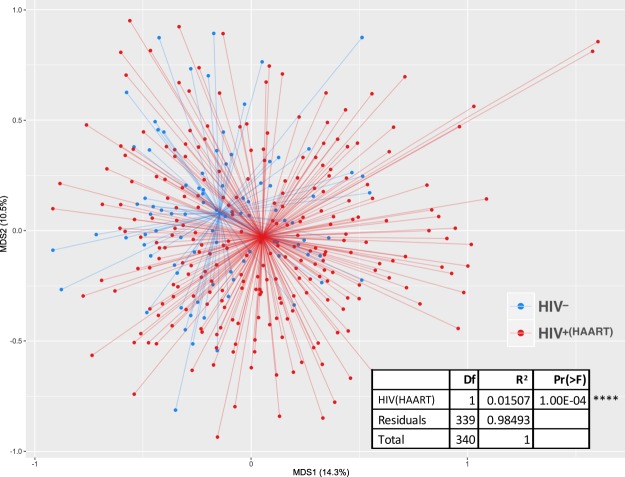
Distance-based analysis of Effects of HIV^+(HAART)^ on microbial community composition without considering possible clinical confounding variables. Non-metric multidimensional scaling ordination of Bray-Curtis dissimilarity between oral microbial communities is shown. The spiders connect the sample points to the centroid of each group. The inset table indicates a PERMANOVA analysis of the difference between groups.

Sequencing of the amplified V1-V3 region of the 16S rRNA gene was performed with the Illumina MiSeq and resulted in a mean of 47,859 raw reads per sample, with a minimum of 684 (not including controls). The results of classification of the sequences are given in Supplementary Dataset 1, subject metadata is in Supplementary Dataset 2, and OTU taxonomy is given in Supplementary Dataset 3. Distance-based community analysis of oral microbial communities showed a difference between the HIV-positive and HIV-negative groups when possible clinical confounding variables were not considered (Fig. 2).

**Figure 3:**
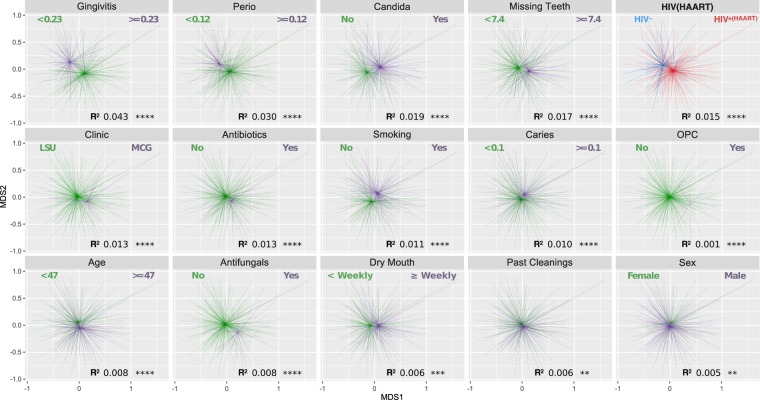
Distance-based analysis of the independent effects of clinical variables on microbial community composition. Only variables that were significant are shown. Non-metric multidimensional scaling ordination of Bray-Curtis dissimilarity was used in independent tests without considering interactions. For visualization of continuous variables samples were grouped based on whether they were greater or less than the mean, and breakpoints are shown. Significance was determined by PERMANOVA.

A number of secondary variables had a significant effect on microbial community composition and were imperfectly balanced by HIV status, and so had the potential to confound comparisons between HIV groups. Figure 3 shows the clinical variables that, when analyzed independently, showed a significant effect on microbial community composition. Supplementary Fig. S1 displaying correlations among clinical variables shows that many were correlated, and some were unbalanced by HIV status. Subsequent distance-based redundancy analysis helped to determine the marginal contribution of these potentially confounding variables.

**Figure 4:**
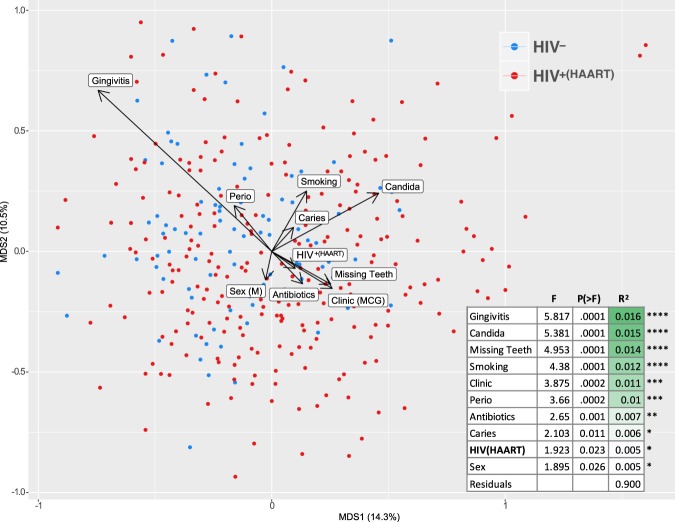
Marginal effects of HIV status and other clinical variables on bacterial communities when interactions are considered. Significant contributory clinical variables were determined by stepwise model selection using distance-based redundancy analysis (dbRDA). Clinical variables that showed a significant effect when interactions were considered are shown. The arrows for the variables show the direction of effect and are scaled by the unconditioned R^2^ value. The table shows marginal effects on the constrained ordination as determined by ANOVA.

Figure 4 shows the results of a distance-based redundancy analysis of oral microbial communities that considered the interactions of potential confounders. All the clinical variables that showed a significant effect on community composition in independent analyses (indicated in Fig. 3) were included in the model selection process. Of the 15 variables entered in the selection process, 10 showed marginal significance, including HIV status. However, as shown, the R^2^ was small for all of them, and the residual was 90%.

**Figure 5:**
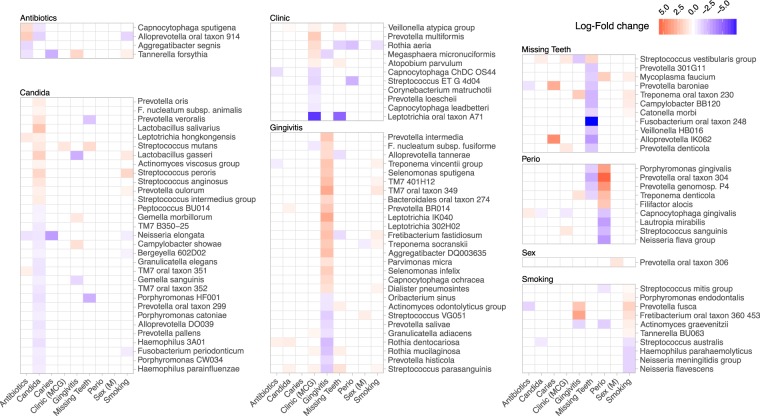
Differential abundance of species for those clinical variables that significantly affected overall microbial community composition. This heat map shows the log-fold change for the 75 species that were significantly differentially abundant by at least one clinical variable. Some species showed differential abundance by more than one clinical variable, but they are shown grouped by the clinical variable with which they were most significantly associated. Although HIV status significantly affected overall community composition, no species was significantly differentially abundant.

Species that were differentially abundant by any of the 10 clinical variables that significantly affected overall microbial community composition are shown in Fig. 5. Multiple species were identified for all of these variables except HIV status, where no significant difference for any species was identified.

Distance-based redundancy analysis was used to examine the effects and interaction of clinical variables within the HIV-positive HAART group. Neither viral load (range 19–1.74 × 10^6^) or CD4 counts (range 1–1661) significantly affected microbial community composition. The effect of viral loads were also analyzed by PERMANOVA comparisons between three groups of subjects: HIV− (n = 89), HIV+/HAART without detectable virus (n = 151), and HIV+/HAART with detectable virus (n = 97). Significant differences were observed between the HIV− group and either of the HIV+ groups (p = 0.001 for either), but not between the HIV+ groups (p = 0.249). Anti-retroviral regimens were categorized by mechanism into reverse transcriptase inhibitors (RTI), integrase inhibitors, non-nucleotide reverse transcriptase inhibitors (NNRTI), and receptor antagonists. Complete data was available for 179 subjects. Most (166) were taking one of 3 regimens: RTI alone (n = 42), RTI with the addition of integrase inhibitors (n = 99) or RTI with the addition of NNRTIs (n = 25). No significant difference in microbial community composition was observed among any of these regimens.

## Discussion

Persons with untreated HIV infection have become increasingly rare in the US over the past decade, resulting in a significant decline in oral disease manifestations throughout the HIV infection continuum. Advances in HAART regimens as well as new guidelines and the earlier initiation of treatment have had a substantial impact, manifested by a reduction in secondary infections and diseases, improved overall health, and a better quality of life^1^. Since the majority of known HIV-positive individuals are undergoing HAART, we focused on comparing them to a well-matched HIV-negative group. Microbial community composition was determined using 16S rRNA gene sequence analysis on the MiSeq platform.

Miseq 2 × 300 base pair reads provided resolution at the level of species, or in a few cases groups of closely related species, when matched to the CORE database^23^. Care was taken to limit and account for potential clinical confounders. The HIV-negative group was recruited from clinic sites that serve clients with high risk behavior for HIV exposure. Extensive metadata on participants’ oral and general health as well as behaviors was collected (summary statistics are shown in Supplementary Table S1). Although the groups were balanced as closely as possible on these variables, differences were observed. A large sample size of 341 individuals was obtained to allow for multivariate analysis to disentangle the effects of potential confounders and separate the true contribution of HIV status. Using this approach, the independent effect of HIV under HAART was found to be statistically significant, but small (p = 0.023, r^2^ = 0.005, Fig. 4).

Approaches to disentangle the effect of the many clinical variables that potentially differ between groups in case-control microbiome studies has received relatively little attention compared to concerns about technical reproducibility in studies. Distance-based redundancy analysis (dbRDA)^22^, a multivariate approach for high-dimensional microbiome data, was used to model the effects of multiple clinical variables. Larger sample sizes than have previously been reported are required, and our larger cohort made it possible to more conclusively address the relationship between HIV status and the oral microbiome. To our knowledge the first published example of constrained ordination as a multivariate analytic approach to microbiome data was to model the contribution of environmental variables in an aquifer ecosystem^24^. It was subsequently used to partition the effects of proinflammatory host gene expression on the oral microbiota in medication related osteonecrosis^25^. Later it was applied to model the gut microbiota and host factors^26^, and subsequently has come into wider use in gut studies.

If only HIV status was considered in the analysis, as shown in Fig. 2, the difference between the HIV positive and negative groups was significant (p = 1.0E-04, r^2^ = 0.015). However, a number of factors such as caries, periodontal disease, and age have all been previously associated with differences in oral microbial community composition, and should be considered. Conceptually the simplest approach would be to balance groups for comparison on every known important variable, but this is seldom feasible in human studies. Multivariate analyses offer a way to sort out independent effects, but require larger sample sizes. For the present study, Fig. 3 shows that many clinical variables showed significant effects on microbial community composition when considered independently, and Fig. 1 shows that a number these were significantly different between the HIV groups. In addition, Supplementary Fig. S1 shows that several clinical variables were correlated with HIV status. This underlying variation is likely to be common in most clinical studies, and clearly indicates the need for deep metadata and a more complex analysis, such as the dbRDA shown in Fig. 4, to tease out the independent effects of variables. Using this approach, the influence of HIV/HAART was statistically significant but smaller in magnitude than some other clinical factors. These included gingivitis, presence of *Candida*, number of missing teeth, current cigarette smoking, clinic site (Augusta or New Orleans), periodontal disease, antimicrobial exposure, and caries (Fig. 4). This list collectively accounted for 8% of the overall variance (r^2^ = 0.08). Previous studies comparing HIV negative and positive groups have generally not considered other clinical variables in the analyses^2,4–6,9–11,15,17,19^. Although oral health measures were often collected^2,11,12,16,17^, either sample sizes were too small to allow meaningful multivariate analysis^2,11,17^ or multivariate analysis was conducted taxon by taxon, allowing potential false discovery errors^12,16^. Only one used dbRDA to calculate the independent effect of periodontal disease and HIV status^13^. Although the sample size was small, with only 10 subjects in the HIV negative group, the results were similar to the current study, with HIV and periodontitis each accounting for around 1% of the total variance.

After multivariate analysis, the residual variation not explained by known variables was large, but consistent with recent observations of the highly personalized nature of the oral microbiota^27^, and presumably reflecting the relative importance of environmental influences^28^.

Seventy-five species were significantly differentially abundant by at least one clinical variable (Fig. 5). No species was identified as significantly differentially abundant between the HIV positive and negative groups, suggesting that small variations across many species accounted for the overall difference observed by dbRDA. Other studies have found changes in bacterial taxa between HIV− and HIV+ subjects, but findings have not been consistently replicated across studies^6,9,11,13,15,17^. In the present study, for gingivitis and periodontal disease, expected species such as *Porphyromonas gingivalis* and other anaerobes previously associated with periodontal diseases emerged, including species of *Prevotella*, *Fusobacterium*, TM7, *Treponema*, *Fretibacterium*, and *Aggregatibacter*. Other studies have considered the effects of periodontal health status in HIV-positive groups^8,14,19^, and also found taxa previously associated with periodontitis. A single *Leptotrichia* species emerged as the biggest driver of the difference between the two study sites, suggesting variation in sampling protocols, or perhaps geographic variation in microbiota.

A positive *Candida* culture, but not oropharyngeal candidiasis (OPC), emerged as a significant contributor to microbial community composition. Positive cultures for *Candida* were associated with bacterial species previously observed in caries. This is consistent with the presence of *Candida* having been associated with caries in children^29^. Approximately half of both groups tested positive for *Candida*, but OPC was observed only in the HIV-positive group, and in only a small subset of the *Candida* positive subjects. The low prevalence of OPC in the HIV-positive cohort (<10%) is consistent with all subjects being on HAART with controlled HIV infection, as therapy has reduced the prevalence of OPC in HIV disease throughout the world^1,30,31^. But this also indicates that OPC continues to be a problem despite HAART, and that the role of fungal species in the oral microbiome of HIV-positive individuals deserves further study. Immune-fungal^32^, fungal-fungal^33^, and bacterial-fungal^21^ interactions have been previously reported. Accordingly, they may be important in disease, and should be more comprehensively analyzed.

Some limitations of the clinical study deserve mention. One is the access to disease management that HIV positive individuals receive in the US, resulting in an HIV positive population whose disease is generally well controlled. The HIV group for this study consisted of HIV-positive adults on HAART for a minimum of six months. The average CD4 cell count was 509 cells/µl (normal range 500–1,500), and the median viral load was below the limit of detection of 20 copies/ml (which we estimated as 19), with only 30 of 252 subjects having >1000 copies/ml. So this group, like the majority of HIV positive individuals in the US, had minimal manifestations of systemic disease, and did not provide a large range of disease severity. Different results might be obtained with a group that included untreated individuals with advanced disease, and further study is warranted. In addition, while it is well documented that periodontal disease is very common in HIV disease^13,34^, the HIV group enrolled in this study had access to dental care at both of the clinical sampling locations. Their periodontal health was similar to that of the HIV-negative group, and they had fewer teeth, probably as a result of this care. Finally, the sampling strategy, a vigorous oral rinse and gargle, was employed because of ease of collection and because it broadly represented all sites in the oral cavity and anterior pharynx. While similar approaches, usually saliva samples or oral swabs, have been widely used in oral surveys, more focused sampling strategies, such as the site-specific subgingival sampling typically used in studies of periodontal status, may have yielded different microbial profiles.

In summary, a multivariate comparison of a large sample of persons with HIV under HAART to an HIV-negative control group showed a complex set of clinical features that influenced oral bacterial community composition, including the presence of HIV under HAART. In addition to establishing an effect of HIV status, these results point to the importance of carefully considering potentially confounding clinical variables in case-control design microbiome studies.

## Methods

### Recruitment

Subjects were recruited through the University Medical Center HIV Outpatient and STD clinics, affiliated with the Louisiana State University Health Sciences Center (LSUHSC) at New Orleans, and Medical College of Georgia (MCG) at Augusta University. Written informed consent was obtained from each participant, and all procedures were conducted in accordance with approvals from the Institutional Review Boards at LSUHSC New Orleans and Augusta University. Inclusion criteria for both groups were age ≥21 years, and having at least 4 remaining teeth. Additional inclusion criteria for the HIV positive group were a confirmed HIV-positive status and at least 6 months on HAART. The HIV-negative group was selectively recruited to match the HIV-positive group on demographic and health factors.

### Clinical data

Participants completed a questionnaire regarding HIV status, race, birth date, gender, daily oral hygiene regimens, alcoholic and non-alcoholic beverage consumption, diet, cigarette use, oral conditions, medications, illegal drug history, and sexual history. Health information was extracted from the medical record. CD4 counts were expressed as average number of cells per microliter. Viral load was expressed as copies per milliliter, and values less than 20 were scored as undetectable. An oral exam conducted by a dentist or dental hygienist included assessment of periodontal disease, caries, oropharyngeal candidiasis (OPC) and other soft tissue lesions. Periodontal assessment included probing at 6 sites per tooth with a CPITN probe. The deepest of the 3 buccal and lingual sites were each recorded as <3.5 mm, ≥3.5 mm but ≤5.5 mm, or ≥5.5 mm. Bleeding upon probing for any site on buccal and lingual was scored as yes or no. Recession on buccal and lingual surfaces was recorded as absent, >0 mm but <3.5 mm, and ≥3.5. Caries scoring included scoring of all surfaces for white spot lesions, active caries, or existing restorations. The diagnosis of OPC was made on the clinical appearance of white curd-like pseudomembranous plaques and/or erythematous atrophic areas on oral mucosa.

### Sample collection

Subjects were instructed to refrain from tooth brushing or using mouthwash within six hours of specimen collection, and receptive oral sex within 24 hours of specimen collection. Sampling was rescheduled as necessary. Oral samples were collected by a supervised 5 mL 30 second swish of sterile saline, alternating between pushing the liquid through the teeth and gums side-to-side and gargling at 5 second intervals. The oral rinse was then expectorated into a sterile 50 ml conical tube and placed on ice. Two volumes (500 µl each) were taken for yeast identification and HPV testing. Qiagen buffer ATL (4 ml) was added to the remaining sample and the suspension was stored at −80 °C. Samples were batched and shipped on dry ice to Ohio State University for analysis. For participants with OPC, swabs from the affected area were collected using the BBL CultureSwab Collection and Transport System (Becton Dickinson) and transported on ice to the lab.

#### Yeast detection

Yeast was identified by plating samples on Chromagar (CHROMagar Microbiology) and incubating for 48 h at 37 °C to observe colony growth. Initial speciation was assigned according to colony color. All rinse samples were screened for asymptomatic colonization by plating 50 ul of each sample. For subjects with OPC, the swab of the infected area was streaked on the plate to confirm yeast colonization.

#### DNA extraction and sequencing

DNA was prepared from oral rinse samples with slight modifications of the QIAamp DNA blood mini kit^35^ (QIAgen, USA). Initially 300 µl of the oral rinse was added to 200 µl of ATL buffer and treated with proteinase K for 2 hours at 56 °C. The samples were then homogenized with 0.25 g of 0.1 mm glass beads in a Mini-Beadbeater-16 (BioSpec Products, USA), before proceeding with the kit instructions. Elutions were performed with 2 × 30 µl of buffer AE heated to 42 °C.

Sequencing libraries were prepared by a two-step procedure based on the Illumina 16S protocol^36^, modified to allow for automation and inclusion of additional index sequences. The 16S gene-specific portions of the primers used to amplify the V1 to V3 region were: (1) A 4:1:1 mixture of AGAGTTTGATYMTGGCTCAG, AGAATTTGATCTTGGTTCAG, AGAGTTTGATCCTGGCTTAG, and AGGGTTCGATTCTGGCTCAG (27F) for the forward end and ATTACCGCGGCTGCTGG for the reverse (534 R). The liquid handling was performed in a Biomek 4000 (Beckman Coulter). First 16S rRNA gene specific primers with 5′ tails were used in an initial amplicon PCR, then dual indices and Illumina-specific adapters were added by a second index PCR with 8 cycles. Oral rinse DNA was adjusted to 5 ng/µl concentration and 2 µl of DNA was amplified in a 25 µl reaction with Accuprime Taq High Fidelity DNA Polymerase (ThermoFisher, USA). The cycling conditions were an initial denaturation at 94 °C for 2 min., 25 cycles of 94 °C for 30 sec., 55° for 30 sec., and 68° for 1 min., with a final extension of 72 °C for 5 min. Amplicons were purified with 20 µl AMPure XP magnetic beads (Beckman Coulter) and used as template in the index PCR reaction which used identical times and temperatures, but for only 8 cycles. We used the extended set of dual indices originally developed by Kozich *et al*.^37^ A minimum of one no template and one mock community control were included on each plate. The mock communities used were either the 21 member mock community provided by BEI Resources or a locally generated mock community with genomes from 7 oral organisms (*Porphyromonas gingivalis*, *Streptococcus mutans, Fusobacterium nucleatum*, *Veillonella parvula*, *Rothia aeria*, *Tannerella forsythia*, *Prevotella nigrescens* and *Neisseria mucosa*). In general, the negative controls gave few reads and the positive controls gave reads that were classified as the bacteria that comprised the mixture, although as others have seen a few 16 S sequences can be generated from reagent controls, and 16 S sequencing of mock communities may not generate the proportions expected. In our case very low numbers of *P. nigrescens* sequences were found. Sequencing was performed on the Illumina MiSeq platform with 2 × 300 base pair reads. The sequenced insert is around 525 bp, and paired end reads overlapped by about 75 bases.

### Sequence data processing

Forward and reverse sequence reads were combined using mothur *make.contigs* with default settings, which performs a Needleman-Wunsch alignment with the parameters match 1, mismatch -1, gapopen -2, and gapextend -1. mothur *screen.seqs* was used to select fragments between 450 and 570 bases with less than 10 ambiguous bases, and mothur *trim.seqs* was used to remove primer sequences^38^. Sequences derived from read pairs with mean base Phred quality less than 28 were removed using a script based on Biopython^39^. Sequences were aligned against the CORE oral 16S rRNA database^23^ using the blastn program, using a php script to re-score the percent identity (avoiding counting ambiguous matches as mismatches). Finally, the best matches over 98% identity were selected to assign taxonomy to the sequences. The data were loaded into a mysql database with sample information via a shell script.

### Statistical analysis

Clinical data were collected and stored in a MySQL database with a custom-built web interface. Caries and periodontal data were aggregated to form indices of oral health. The gingivitis index was calculated as the percentage of sites with bleeding on probing; the caries index as the percent of all surfaces that were decayed (white spots were scored as 0.5 and cavitated lesions as 1); the restoration index as the percentage of restored surfaces; and the periodontal health index as the percentage of sites with PD ≥ 3.5 mm.

Spearman’s rho correlations between clinical variables were calculated using the rcorr function in the Hmisc package in R. Balancing of the clinical variables by HIV status was determined by Wilcox rank sum test for continuous variables and Fisher’s exact test for categorical variables.

Non-metric multidimensional scaling of Bray-Curtis dissimilarities was performed using the metaMDS function from the vegan package in R. For confounder analysis, the independent contributions of clinical variables to microbial community composition were determined with PERMANOVA, a non-parametric permutation-based analysis of variance (adonis function in vegan). The direction of the effect of clinical variables was modeled using the envfit function in vegan, and the magnitude was scaled to the effect size (R^2^) determined by PERMANOVA.

Distance-based redundancy analysis (db-RDA) was done using capscale (vegan). Variables were included in a stepwise db-RDA model selection process (ordiR2step, vegan). The marginal significance of the remaining variables was tested by a permutation-based ANOVA for constrained ordinations (anova.cca, vegan). Only the clinical variables that were significant contributors to the variation in microbial community composition after adjusting for multiple comparisons (Benjamini & Hochberg) were entered into the model selection for the db-RDA ordination.

Differential species abundance was performed by fitting each species to a generalized linear model. Only those species with mean prevalence ≥0.1%, and only the 10 clinical variables that were significant contributors to the variation in microbial community composition by the RDA analysis were included in this analysis. For each species, count data was fitted to a multiple linear regression by clinical variables using a negative-binomial, zero-inflated distribution (glmmTMB). The significance of resulting model terms was assessed with the Wald test, and species p-values were adjusted for the clinical variable using Benjamini-Hochberg.

## Supplementary information

Supplementary Info

Dataset 1

Dataset 2

Dataset 3

## Data Availability

Sequence files have been deposited in the NCBI Sequence Read Archive (SRA) under BioProject ID PRJNA530161. A pre-release link for reviewers can be accessed here: https://dataview.ncbi.nlm.nih.gov/object/PRJNA530161?reviewer=4psogcg4k51g2rkfd640q5mc3r.
