# Supplementary material for: Significant effect of HIV/HAART on oral microbiota using multivariate analysis
Source: Sci Rep. 2019 Dec 27;9:19946. doi: 10.1038/s41598-019-55703-9 (PMC6934577; doi:10.1038/s41598-019-55703-9)
Supplement: Supplementary file 1 — Supplementary Info [file 41598_2019_55703_MOESM1_ESM.pdf]

SUPPLEMENTARY INFORMATION: Significant effect of HIV/HAART on oral microbiota using multivariate analysis

Ann L. Griffen[a,#], Zachary A. Thompson[b], Clifford J. Beall[b], Elizabeth A. Lilly[c], Carolina Granada[d], Kelly D. Treas[c], Kenneth R. DuBois III[c], Shahr B. Hashmi[b], Aubrey E. Gilliland[c], Jose A. Vazquez[d], Michael E. Hagensee[e], Eugene J. Leys[b], Paul L. Fidel, Jr.[c]

Supplementary Table S1. Comparison of clinical status for the HIV^{-} and HIV$^{+(ART)}$ groups

Binary variable	Levels		HIV-	HIV+/HAART	Signif.
Candida culture +	No | Yes		34 | 55 (62%)	109 | 143 (57%)	
Current antibiotics	No | Yes		84 | 5 (5.6%)	189 | 63 (25%)	****
Current antifungals	No | Yes		86 | 3 (3.4%)	204 | 47 (19%)	***
OPC	No | Yes		89 | 0 (0%)	235 | 17 (6.7%)	**
Clinic	LSU | MCG		81 | 8 (9%)	205 | 47 (19%)	*
Sex	Female | Male		28 | 61 (69%)	70 | 182 (72%)	
Race	African-American | White		57 | 29 (34%)	202 | 41 (17%)	**
Cleanings in past year	None | Any		51 | 35 (41%)	97 | 147 (60%)	**
Floss frequency	Less than weekly | Weekly or more		44 | 41 (48%)	116 | 130 (53%)	
Brush frequency	Less than daily | Daily or more		2 | 87 (98%)	16 | 236 (94%)	
Mouthwash frequency	Less than weekly | Weekly or more		40 | 49 (55%)	85 | 167 (66%)	
Dry mouth	Less than weekly | Weekly or more		50 | 36 (42%)	108 | 142 (57%)	*
Soda	Diet | Regular		21 | 68 (76%)	86 | 166 (66%)	
Sugar diet	Normal | High sugar		80 | 9 (10%)	234 | 18 (7.1%)	
Currently smoking	No | Yes		39 | 50 (56%)	125 | 127 (50%)	
Oral Sex	No | Yes		57 | 32 (36%)	194 | 54 (22%)	*
Daily alcohol use	No | Yes		66 | 22 (25%)	163 | 89 (35%)	
Past recreational drug use	No | Yes		47 | 42 (47%)	159 | 82 (34%)	*
Current marijuana use	No | Yes		69 | 6 (8%)	197 | 26 (12%)	
Quantitative variable	HIV-		HIV+/HAART		Signif.
	Mean	CI +/-	Mean	CI +/-	
Age	40.5	2.7	49.0	1.3	****
Missing teeth	4.6	1.3	8.5	0.93	****
Gingivitis	0.29	0.046	0.21	0.027	**
% Attachment level	0.14	0.047	0.11	0.021	
% Decayed surfaces	0.10	0.025	0.10	0.017	
% Restored surfaces	0.054	0.017	0.074	0.012	

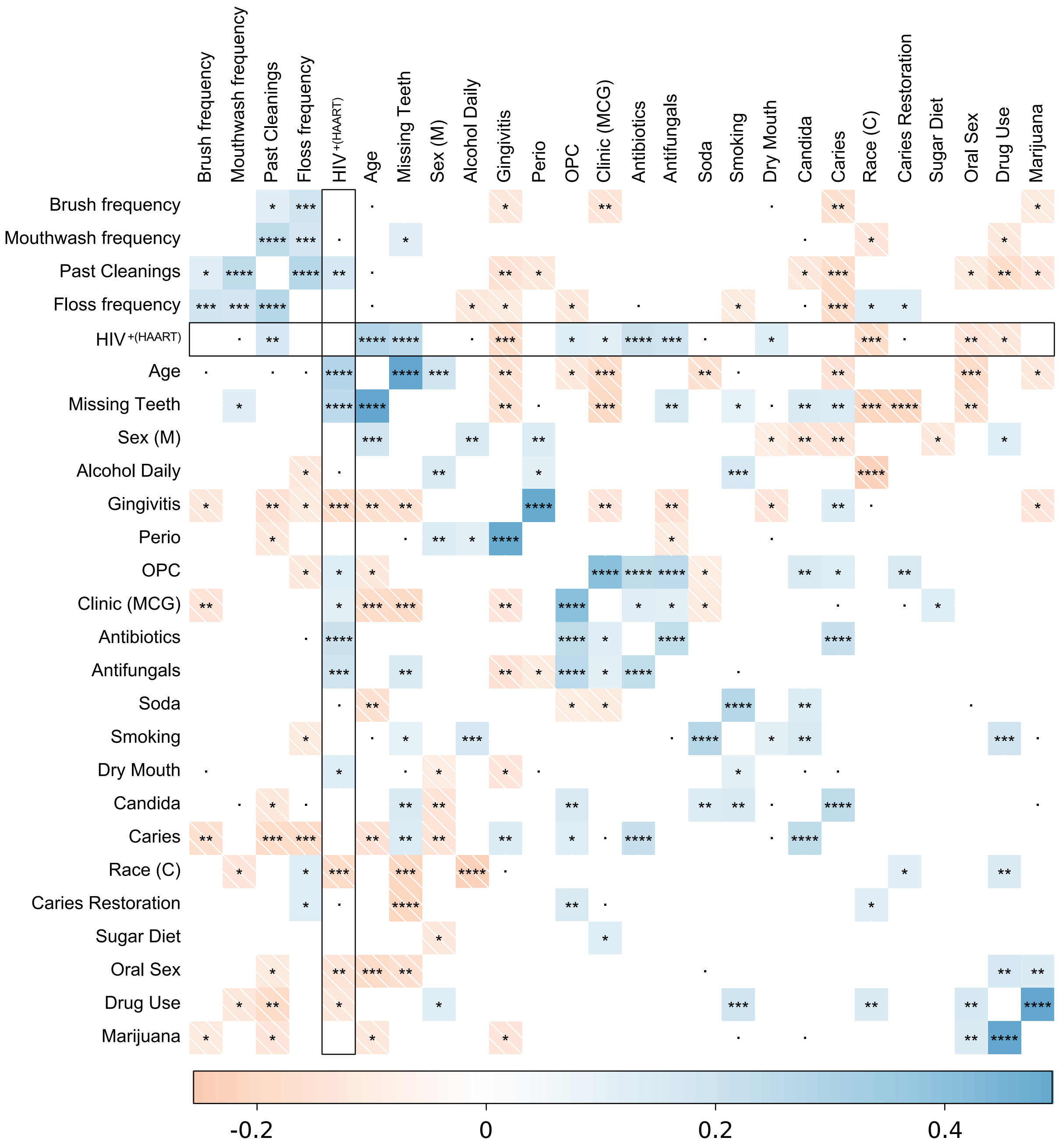

Supplementary Figure S1. Correlations between clinical variables measured. Many secondary clinical variables were correlated. Spearman correlation coefficients between all clinical variables are shown. Asterisks indicate levels of significance: *, p<0.05; ** p<0.01; ***, p<0.001; ****, p<0.0001.
